# Novel deep learning framework for detection of epileptic seizures using EEG signals

**DOI:** 10.3389/fncom.2024.1340251

**Published:** 2024-03-21

**Authors:** Sayani Mallick, Veeky Baths

**Affiliations:** ^1^Cognitive Neuroscience Laboratory, Department of Electrical and Electronics Engineering, BITS Pilani, KK Birla Goa Campus, Pilani, Goa, India; ^2^Cognitive Neuroscience Laboratory, Department of Biological Sciences, BITS Pilani, KK Birla Goa Campus, Pilani, Goa, India

**Keywords:** LSTM, GRU, convolutional neural networks, deep learning, machine learning, automated framework, epilepsy

## Abstract

**Introduction:**

Epilepsy is a chronic neurological disorder characterized by abnormal electrical activity in the brain, often leading to recurrent seizures. With 50 million people worldwide affected by epilepsy, there is a pressing need for efficient and accurate methods to detect and diagnose seizures. Electroencephalogram (EEG) signals have emerged as a valuable tool in detecting epilepsy and other neurological disorders. Traditionally, the process of analyzing EEG signals for seizure detection has relied on manual inspection by experts, which is time-consuming, labor-intensive, and susceptible to human error. To address these limitations, researchers have turned to machine learning and deep learning techniques to automate the seizure detection process.

**Methods:**

In this work, we propose a novel method for epileptic seizure detection, leveraging the power of 1-D Convolutional layers in combination with Bidirectional Long Short-Term Memory (LSTM) and Gated Recurrent Unit (GRU) and Average pooling Layer as a single unit. This unit is repeatedly used in the proposed model to extract the features. The features are then passed to the Dense layers to predict the class of the EEG waveform. The performance of the proposed model is verified on the Bonn dataset. To assess the robustness and generalizability of our proposed architecture, we employ five-fold cross-validation. By dividing the dataset into five subsets and iteratively training and testing the model on different combinations of these subsets, we obtain robust performance measures, including accuracy, sensitivity, and specificity.

**Results:**

Our proposed model achieves an accuracy of 99–100% for binary classifications into seizure and normal waveforms, 97.2%–99.2% accuracy for classifications into normal-interictal-seizure waveforms, 96.2%–98.4% accuracy for four class classification and accuracy of 95.81%–98% for five class classification.

**Discussion:**

Our proposed models have achieved significant improvements in the performance metrics for the binary classifications and multiclass classifications. We demonstrate the effectiveness of the proposed architecture in accurately detecting epileptic seizures from EEG signals by using EEG signals of varying lengths. The results indicate its potential as a reliable and efficient tool for automated seizure detection, paving the way for improved diagnosis and management of epilepsy.

## 1 Introduction

Epilepsy is a prevalent chronic neural disorder caused by irregular electrical discharges in the brain, known as seizures. It is a neurological disorder affecting millions of people worldwide. Epileptic seizures can vary in severity and duration and can cause a wide range of symptoms, including loss of consciousness, convulsions, muscle spasms and sensory disturbances. Seizures are caused due to uncontrolled electrical discharges in a group of neurons in the brain which leads to disruption of the brain function (Gandhi et al., 2011). The accurate and timely detection of seizures is one of the critical challenges faced in managing epilepsy today.

Electroencephalography (EEG) is used widely in the detection of epileptic seizures. It is a non-invasive recording of brain activity which records the voltage fluctuations resulting from the ionic flow of neurons in the brain, reflecting the brain's bio-electric activity. Neurologists make inferences from the EEG signals by visual inspection which is a laborious and time-intensive process requiring skimming through hundreds of hours of EEG recordings. This method of detection of epileptic seizures from EEG signals is highly dependent on neurologists' expertise. Therefore, attempts have been made to automate the process of epileptic seizure detection from EEG signals using machine learning and deep learning.

Researchers have made efforts to use machine-learning techniques for the detection of epileptic seizures by extracting handwritten features in the time domain and frequency domain and often combining both these domains together. Most of the works in the field of seizure detection involve two stages. The first stage involves feature extraction in the time domain, frequency domain, or time-frequency domain. Researchers have used signal transformations like Fast Fourier Transform, Short-Time Fourier Transform, and wavelet transforms for frequency domain analysis for extracting features from EEG signals. The second stage involves using the extracted hand-crafted features for classifying the EEG signals using classifiers. However, the performance of these models is highly dependent on the ability of the experts to handcraft the features.

The effectiveness of time-frequency analysis in categorizing EEG segments for epileptic seizures has been illustrated previously (Tzallas et al., 2007). The researchers utilized an artificial neural network as the classifier attaining an 89% accuracy for the five-class classification problem. Orhan et al. (2011) employed the k-means algorithm to perform clustering on wavelet coefficients followed by multilayer perceptron neural networks.

Miltiadous et al. (2021) utilized a five-level Discrete Wavelet Transform (DWT) to decompose EEG signals into sub-bands to extract features and train a Random Forest Classifier. Raghu et al. (2019) proposed a novel feature named successive decomposition index for automated seizure detection.

Deep learning techniques offer automated detection of epileptic seizures from EEG signals, removing the necessity for manual feature engineering, as convolutional neural networks and recurrent neural networks can learn hierarchical representations of data through interconnected layers of neurons. The features extracted by deep learning models from non-stationary biomedical signals like EEG signals are known to be more robust than hand-crafted features (LeCun and Bengio, 1995).

Standard recurrent neural networks have been used in several studies for the detection of seizures. Recurrent neural networks are suitable for time-sequence data like EEG signals. However, standard recurrent neural networks are subject to the problem of vanishing gradients and exploding gradients (Bengio et al., 1994). To solve the problem of vanishing and exploding gradients, long short-term memory and gated recurrent units can be used. There have been many recent advances in the detection of epileptic seizures from EEG signals.

Ullah et al. (2018) proposed an ensemble of pyramidal one-dimensional convolutional neural network models. The author has achieved an accuracy of ~99% on the Bonn University dataset for the classification of EEG signals into normal, interictal and ictal. Roy et al. (2019) proposed a new architecture called Chrononet which consists of multiple 1-D convolution layer. Hussein et al. (2019) proposed a robust architecture for detecting epileptic seizures using Long Short-Term Memory (LSTM) network. Thara et al. (2019) proposed a model for seizure detection as well as seizure prediction using stacked LSTM and Bi-LSTM and an accuracy of 99% on the Bonn University dataset. Minasyan et al. (2010) used neural network and fuzzy function with a combination of principal component analysis for seizure detection and achieved an accuracy of 97.64%.

Convolutional Neural Networks have also been used to detect the spikes in the EEG data (Johansen et al., 2016). Acharya et al. (2018) applied a 13-layer deep CNN algorithm to an iEEG Freiburg dataset to detect normal, pre-ictal and seizure classes. They achieved an accuracy, specificity, and sensitivity of 88.67%, 90.00%, and 95.00%, respectively.

Duan et al. (2019) used CNN-based spectral sub-band features related to correlation coefficients of electrodes for EEG segments of duration 1 s, 2 s, and 3 s. They achieved an accuracy of 94.8%, sensitivity of 91.7%, and specificity of 97.7%. Aarabi et al. (2009) achieved an accuracy of 93%, F1 score of 95%, and a sensitivity of 91% using BNN on the Freiburg dataset.

In this work, a novel deep learning framework incorporating the use of units of 1D-CNN, Bidirectional LSTM and Average Pooling layer has been proposed for the classification of seizure and non-seizure EEG signals as well as multiclass classifications into different stages of seizures using the Bonn dataset. The features extracted by the units of 1D-CNN, Bi-LSTM and Average Pooling layer are passed to the Flatten layer and subsequently, Dense layers are used. The proposed models have also been developed with Bidirectional GRUs instead of Bidirectional LSTMs and their performances have been compared.

Epilepsy is a neurological disorder with various seizure types and manifestations. Therefore, it is necessary to be able to distinguish between various types of seizure signals is extremely important and therefore, classification into four and five-class classifications provides a more granular diagnosis support. Few studies have addressed the problem of classification of EEG signals into different stages. In this study, we have studied five classification problems: binary, ternary, four class, and the five class classification problem, and achieved significant improvements in the performance measures as compared to previous studies.

The proposed work has shown to be effective in the detection of epileptic seizures using EEG signals in the presence of artifacts as well as in ideal conditions. It has been experimented on EEG signals of different durations to determine its effectiveness by varying the number of filters used in the convolutional layers, the number of units used in the Bi-LSTM, and the number of Dense layers and their parameters. We report that it has obtained significant improvements in the performance measures, namely accuracy, specificity, sensitivity and F1 score.

While there have been many studies and research on the classification of EEG signals to detect epileptic seizures, very few studies have proposed a novel architecture that has achieved considerable improvements in the results for the classification of EEG signals of varying lengths as well as in the presence and absence of artifacts. One of the major contributions of this study is that it addresses the problem of binary as well as multi-class classification of EEG signals of varying lengths in the presence and absence of artifacts and achieves improvements over previous studies. This study also examines the inference time as well as the training times of the proposed models for each classification.

## 2 Materials and methods

### 2.1 Dataset

The dataset used in this study is of EEG signals obtained from the Bonn University, Germany (Andrzejak et al., 2001). This dataset has been used extensively in the detection of epileptic seizures. The dataset consists of 500 segments of EEG recordings divided evenly into five sets. These signals are recorded from a 128-channel amplifier using a 12-bit analog-to-digital converter. Each set has a total of 100 single-channel EEG signals with 4,097 sample points per channel. Every signal has a duration of 23.6 seconds and a sampling frequency of 173.61 Hz. The dataset consists of five sets of EEG signals: A, B, C, D and E. Set A and set B are recorded from the scalp of five healthy subjects with their eyes open and closed, respectively. The 10–20 standard electrode placement was used. The set C, D, and E are EEG signals collected from five epileptic patients. The set D consists of EEG signals recorded from the epileptogenic zone. Set C consists of EEG signals recorded from the opposite hemisphere's hippocampus formation during the seizure-free intervals, known as the inter-ictal state. Set E consists of true seizure waveforms recorded during the ictal stage.

To perform the experiments, we have studied the following five classifications as specified in Table 1.

**Table 1:** Classification type and set combination used in the study.

**Classification type**	**Data combination**
Two class classification	A vs. E
Two class classification	B vs. E
Three class classification	AB vs. CD vs. E
Four class classification	AB vs. C vs. D vs. E
Five class classification	A vs. B vs. C vs. D vs. E

### 2.2 Proposed framework

Our proposed architecture incorporates the use of 1D-CNNs, Bidirectional LSTMs, and Average Pooling Layer as a unit. This unit is repeatedly used depending on the classification problem. Each unit is separated by a Dropout layer to prevent overfitting of the models. The EEG signals from the Bonn dataset after preprocessing, are fed as input to the 1D-CNN layers. The 1D-CNN layer performs convolution operations on the input data, extracting relevant features from the sequential information. The convolutional filters learn to detect specific patterns in the EEG signals that may indicate seizures. The Bidirectional LSTM layer captures the long-term dependencies and temporal dynamics in the EEG signals. It processes the input data in both forward and backward directions, allowing the model to effectively analyze the sequential information. The LSTM units maintain the memory of past information, enabling the model to retain important context while evaluating the EEG signals. The Average Pooling layer reduces the dimensionality of the extracted features by performing down-sampling.

Dropout layers inserted between the repeated units of the architecture randomly deactivates a fraction of neurons during training, preventing overfitting and enhancing the model's generalization ability (Srivastava et al., 2014). The Flatten layer reshapes the output from the previous layers into a vector form, preparing it for the subsequent Dense layers. The Dense layers are fully connected layers that further process the learned features. They perform computations on the flattened vector to generate the final classification or prediction results.

**Figure 1:**
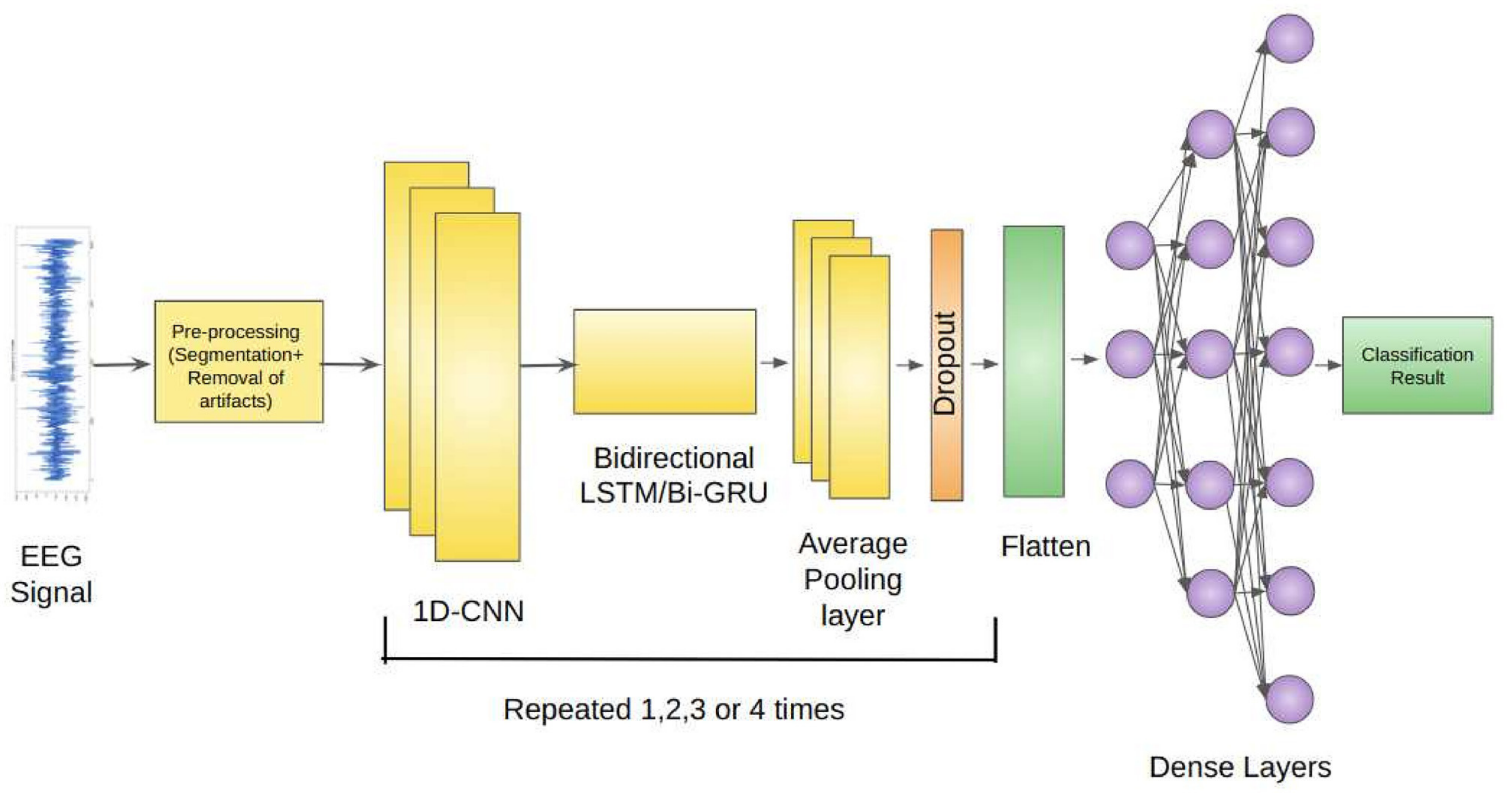
Proposed framework for the detection of epileptic seizures from EEG signals.

Figure 1 depicts the proposed framework architecture.

We have tested our proposed architecture for EEG signals of duration 23.6, 11.8, and 1 s in the following six studies consisting of the following types of input EEG signals:

1. Study 1: Unfiltered EEG signals of duration 23.6 s.
2. Study 2: Filtered EEG signals of duration 23.6 s.
3. Study 3: Unfiltered EEG signals segmented into duration of 11.8 s.
4. Study 4: Filtered EEG signals segmented into duration of 11.8 s.
5. Study 5: Unfiltered EEG signals segmented into duration of duration 1 s.
6. Study 6: Filtered EEG signals segmented into duration of duration 1 s.

Study 1.1 used Bidirectional LSTMs, and Study 1.2 used Bidirectional GRUs. This convention is followed throughout the paper where first part corresponds to models with Bi-LSTM and second part corresponds to Bi-GRU.

#### 2.2.1 Pre-processing

**Figure 2:**
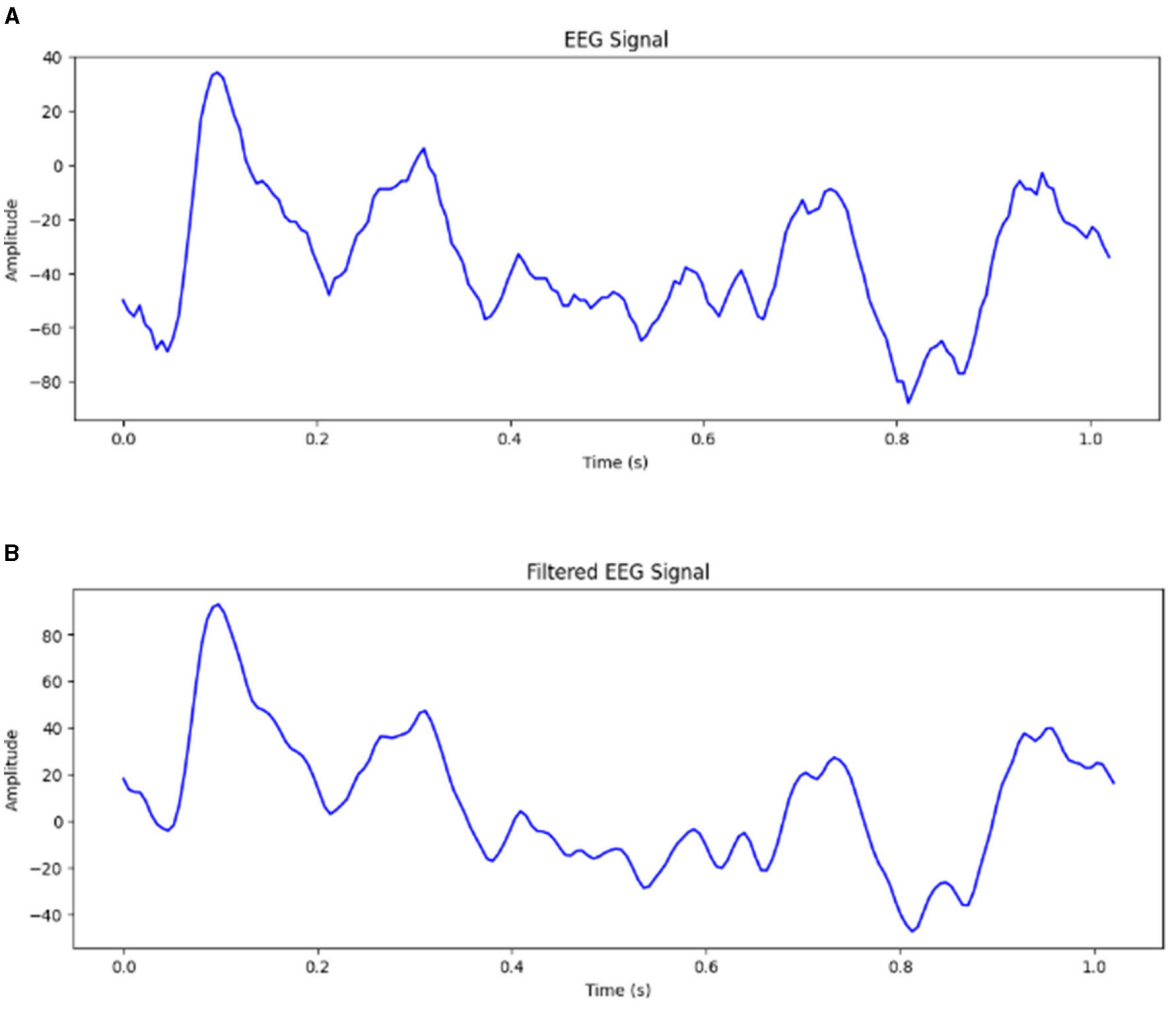
**(A)** EEG signal before filtering. **(B)** EEG signal after filtering.

The raw EEG signals obtained from the Bonn dataset are contaminated with noise EEG signals may be contaminated with many artifacts. The frequency range of EEG recordings in the Bonn dataset is 0–86.8 Hz. Frequencies higher than 50 Hz are considered noise. It can be difficult for the model to extract meaningful features and capture the underlying patterns if the input signals contain too much noise. It has been observed that using EEG signals contaminated with noise has resulted in a drop of accuracy by 10% (Abualsaud et al., 2015). Therefore, it is important to preprocess the input signals to remove the noise before feeding them into the model. The proposed framework has been tested for both unfiltered raw EEG signals and filtered EEG signals. For Study 2, 4 and 6, the signals obtained from the Bonn dataset are passed through a zero-phase band-pass Butterworth filter of order two which limits the frequency content of the signals to a range of [0.5, 50] Hz. Figure 2 shows the EEG signal of duration 1 s before and after passing through band-pass filter.

#### 2.2.2 Convolutional neural networks

Convolutional Neural Networks have shown promising results over the past decade in various fields related to pattern recognition (Albawi et al., 2017). Time series data can be considered a one-dimensional grid formed by regular sampling on the time axis. 1D-CNNs are intrinsically suitable for processing biological signals such as EEG for epileptic seizure detection (Shoeibi et al., 2021). Convolutional layers extract relevant features from the input data by applying a set of filters to the input. Convolutional Neural Network has the characteristic of sparse interaction, resulting in fewer parameters to be stored and simplifying calculations.

#### 2.2.3 Long short-term memory

Long Short-Term Memory (Hochreiter and Schmidhuber, 1997) is a type of Recurrent Neural Network architecture widely used for sequential data processing tasks such as speech recognition, natural language processing and time series prediction.

Unlike traditional RNNs, LSTMs are designed to capture long-term dependencies in the input sequence. This is achieved through a memory cell that can store and selectively retrieve information over time and three gates that regulate the flow of information into and out of the cell.

**Figure 3:**
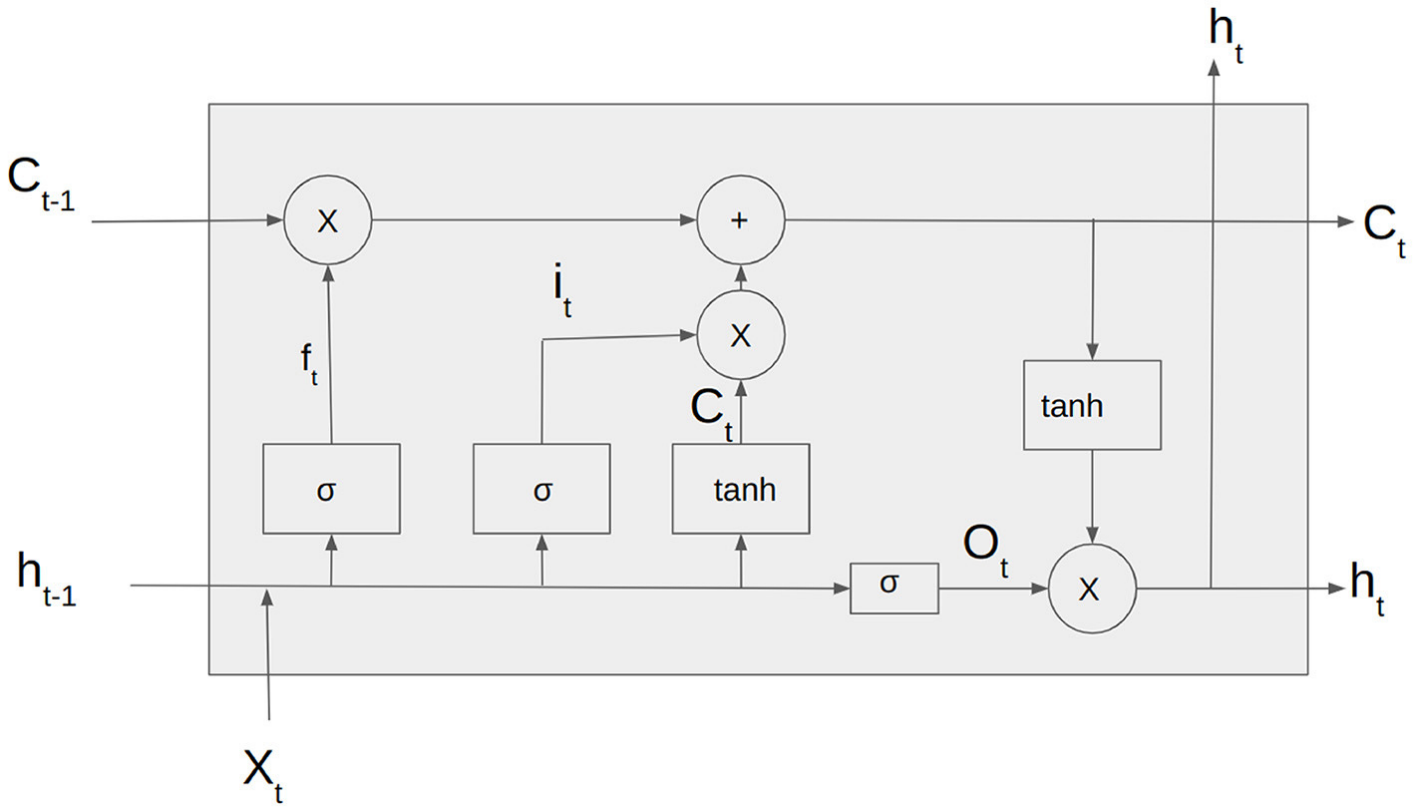
Architecture of LSTM cell.

The Figure 3 depicts the input vector, *x*_*t*_, the input gate *i*_*t*_. *f*_*t*_ is the forget gate vector, *O*_*t*_ is the output gate vector, *h*_*t*_ is the output of the LSTM cell at the time step t and *C*_*t*_ is the current cell state. The LSTM cell consists of three gates: forget gate, input gate, and output gate. The forget gate is responsible for deciding whether the information in the given data sample should be forgotten or retained.

This work uses Bidirectional LSTM, which processes the input in both forward and backward directions (Nagabushanam et al., 2020). This is beneficial when handling EEG signals of long durations.

#### 2.2.4 Gated recurrent units

Gated recurrent units are a form of recurrent neural network similar to LSTMs. They have the additional advantage of fewer trainable parameters (Chung et al., 2014). A GRU consists of a reset gate, an update gate, and a candidate activation function. The reset gate controls how much of the previous hidden state is forgotten, while the update gate controls how much of the new input is incorporated into the current hidden state. The candidate activation function calculates the new hidden state based on the input and the previous hidden state, and this new hidden state is then passed to the next time step.

#### 2.2.5 Pooling layer

The main idea of using a pooling layer is to downsample to reduce the complexity for further layers in the framework (Albawi et al., 2017). In our work, we have used the Average Pooling layers for the following reasons:

- Reducing the feature dimension: Conv1D and LSTM layers can generate high-dimensional feature maps that can be computationally expensive to process. AvgPool1D layers can be used to reduce the feature dimension and simplify the computations, making the model more efficient.
- Improving the generalization of the model: AvgPool1D improves the generalization of the model and helps in reducing overfitting.

#### 2.2.6 Dropout

Dropout is a regularization technique used in neural networks to prevent overfitting (Srivastava et al., 2014). It randomly sets a certain proportion of input units to zero during each training iteration, preventing the network from relying too much on any single feature. In this work, we have used Dropout layers to prevent overfitting of the models to the training data.

#### 2.2.7 Flatten

We use the Flatten layer in neural networks to convert a multidimensional tensor into a one-dimensional tensor, which can then be fed into a fully connected layer. This is necessary when we want to use a fully connected layer to classify the output of a convolutional or recurrent layer.

#### 2.2.8 Fully connected layers

Fully connected layers have been used after the feature extraction using 1D-CNN and Bi-LSTM/ Bi-GRU unit to learn the non-linear relationships between the extracted features and the output. In this work, non-linear activation functions like ReLU are applied to the resulting vector, allowing the network to learn the complex relationships between the features and the output.

### 2.3 Training and validation details

For binary classifications, 200 EEG signal samples were used, while for multi-class classifications, all 500 samples from the Bonn dataset were utilized.

Our proposed models were trained by optimizing the categorical cross-entropy cost function with Adam optimizer. The Adam optimizer has been used because it combines the advantages of the AdaGrad and RMSProp algorithms. The optimizer enhances computational efficiency during the training of the Bi-LSTM and Bi-GRU model (Nagabushanam et al., 2020).

The proposed models are trained and evaluated using five-fold cross-validation. The EEG signals are randomly divided into five parts of equal size. Four-folds have been used for training and one-fold for testing.

In Study 1 and 2, 160 EEG signals of 23.6 s are used for training, with 40 signals for testing in each fold. For the three, four, and five class classifications, 400 EEG signals of 23.6 s are used for training, and 100 signals for testing. In Study 3 and 4, EEG signals are segmented into 11.8 s non-overlapping segments, creating 1,000 signal segments, with 800 used for training and 200 for testing the models.

## 3 Study

In our work, we conducted experiments using various models, varying different parameters such as the number of filters applied and kernel size of the filters in the convolutional layers, number of LSTM/GRU units and dropout size. In this work, the proposed models incorporate the parameters that yielded the best performance in each classification in each study. The Bi-LSTM and Bi-GRU models shared identical parameter settings for each classification within each study. The proposed models follow the proposed architecture incorporating the use of 1D-CNN, Bi-LSTM/GRU and Average Pooling Layer as a unit followed by the Flatten and Dense layers.

To evaluate the robustness of our proposed approach toward noise in the EEG signals, we have used unprocessed raw EEG signals and preprocessed signals from the Bonn dataset as input to the model. To preserve the long-term temporal dependencies we employ Bidirectional LSTMs or Bidirectional GRUs instead of unidirectional LSTMs/GRUs. This simulates real-life scenarios where the EEG signals can be of varying durations and can be corrupted with artifacts and white noise.

Table 2 shows the details of the proposed models for each classification. 1D-CNN(i) and LSTM(i) indicate the convolutional layer and the LSTM layer in the (*i*th) unit.

**Table 2:** Architecture for the proposed models.

**Classification**	**CNN (1)**	**LSTM (1)**	**CNN (2)**	**LSTM (2)**	**CNN (3)**	**LSTM (3)**	**CNN (4)**	**LSTM (4)**
**Study 1**								
A–E	32	16	16	8	–	–	–	–
B–E	32	32	16	16	–	–	–	–
AB–CD–E	64	8	128	4	–	–	–	–
AB–C–D–E	128	32	64	16	32	8	–	–
A–B–C–D–E	128	32	64	16	32	8	–	–
**Study 2**								
A–E	4	4	8	8	-	–	–	–
B–E	16	16	8	8	–	–	–	–
AB–CD–E	256	128	64	64	32	32	–	–
AB–C–D–E	64	64	32	32	16	16	–	–
A–B–C–D–E	128	64	64	32	32	16	–	–
**Study 3**								
A–E	256	64	64	32	32	16	–	–
B–E	256	64	64	32	32	16	–	–
AB–CD–E	32	16	16	8	8	4	–	–
AB–C–D–E	256	32	128	32	64	16	32	8
A–B–C–D–E	64	128	128	64	256	32	–	–
**Study 4**								
A–E	16	8	32	16	–	–	–	–
B–E	32	32	64	64	128	128	–	–
AB–CD–E	150	100	120	50	100	25	–	–
AB–C–D–E	32	64	64	32	128	16	256	8
A–B–C–D–E	32	16	64	32	128	64	–	–
**Study 5**								
A–E	128	64	64	32	16	16	–	–
B–E	128	64	64	32	32	16	–	–
AB–CD–E	64	32	32	16	–	–	–	–
AB–C–D–E	128	128	64	64	–	–	–	–
A–B–C–D–E	300	300	150	150	–	–	–	–
**Study 6**								
A–E	16	8	–	–	–	–	–	–
B–E	32	16	–	–	–	–	–	–
AB–CD–E	16	8	32	16	64	32	–	–
AB–C–D–E	64	64	128	128	–	–	–	–
A–B–C–D–E	256	256	128	128	–	–	–	–

### 3.1 Study 1: unfiltered EEG signals of duration 23.6 s

The Bonn dataset consists of EEG signals of length 23.6 s, each corresponding to 4,097 sampling points. In Study I, the full-length raw EEG signals have been fed to the proposed architecture. The batch size was set to 64.

All the convolutional layers in the proposed work employ a kernel size of 2, stride of 1, and valid padding. The classification study for A–E used 100 units in the Dense layer, while the B–E model used 3 Dense layers with 100, 50, and 10 units respectively. The classification of AB–CD–E used 3 Dense layers with 80, 40 and 20 units, while AB–C–D–E used 1 Dense layer with 32 units and A–B–C–D–E employed three Dense layers with 100, 50, and 25 units respectively. The A–E classification model has been trained for 50 epochs, while the B–E classification model has been trained for 100 epochs. The other models have been trained for 150 epochs.

### 3.2 Study 2: filtered EEG signals of duration 23.6 s

In Study 2, all five sets of raw EEG signals obtained from the Bonn dataset are passed through a zero-phase band-pass Butterworth filter of order two which limits the frequency content of the signals to a range of [0.5, 50] Hz.

A batch size of 64 and a dropout rate of 0.1 was used in this study. A single Dense layer with 100 units is utilized for the binary classifications, while AB–CD–E and A–B–C–D–E classifications utilize Dense layers with 200 and 50 units respectively. Convolutional layers in A–E, B–E, and AB–CD–E classifications have filters with a kernel size of 2, whereas AB–C–D–E and A–B–C–D–E classifications use filters with a kernel size of 4. All convolutional layers employ valid padding and a stride of 1. The A–E model is trained for 50 epochs, B–E for 100 epochs, and the other models for 150 epochs each.

### 3.3 Study 3: unfiltered EEG signals of duration 11.8 s

In Study 3, the raw EEG signals from the Bonn dataset are segmented into signals of duration 11.8 s, corresponding to 2,048 sampling points as input to the proposed models. No other pre-processing of the signals is carried out prior to the training.

The models were trained for 150 epochs, with 0.1 dropout rate. The A–E and B–E models use two Dense layers with 200 and 50 units. AB–CD–E model uses Dense layers with 100 and 40 units while AB–C–D–E model uses two Dense layers with 200 and 100 units.

### 3.4 Study 4: filtered EEG signals of duration 11.8 s

**Figure 4:**
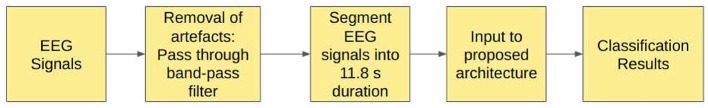
Pipeline of Study 4.

In Study 4, the EEG signals from the Bonn dataset are passed through the band-pass Butterworth filter to remove the noise and artifacts present in the raw EEG signals. These signals are then segmented into signals of duration 11.8 s, corresponding to 2,048 sampling points before being fed into the proposed model, as shown in the Figure 4.

The batch size is chosen to be 64. The dropout of 0.1 is used. The A–E classification model uses three Dense layers of 100, 50, and 10 units, respectively. The B–E classification model uses two Dense layers of 100 and 50 units. The model for AB–CD–E classification uses a Dense layer with 100 units. The AB–C–D–E classification model has a single Dense layer with 200 neurons, whereas the model for A–B–C–D–E classification uses a single Dense layer with units.

### 3.5 Study 5: unfiltered EEG signals of duration 1 s

In Study 5, EEG signals were segmented into 1 s durations, each with 178 sampling points, and fed to the proposed models.

The AB–CD–E classification model employed Dense layers with 100 and 20 units, while the AB–C–D–E classification model employed Dense layers with 100 and 40 units. The AB–C–D–E classification model employed convolutional layers with a kernel size of 4, stride of 1, and valid padding. Conversely, the A–B–C–D–E classification model employed convolutional layers with a kernel size of 8. A dropout rate of 0.2 was applied specifically to the A–B–C–D–E classification model, while a dropout rate of 0.1 was used for the other models.

### 3.6 Study 6: filtered EEG signals of duration 1 s

**Figure 5:**
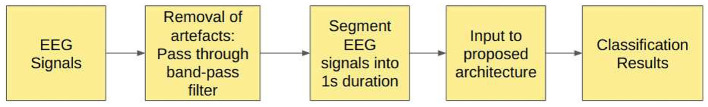
Pipeline of Study 6.

In Study 6, the raw EEG signals are passed through the band-pass Butterworth filter to remove noise and artifacts and then segmented into durations of 1 s each, corresponding to 178 sampling points. Figure 5 illustrates the steps involved in this study.

The proposed model for binary classification incorporates a 1D-CNN and Bi-LSTM/GRU without Dropout. Dense layers consist of 100 and 10 units.

For AB–CD–E classification, convolutional layers with kernel size 2, stride 1, and valid padding were employed. Three Dense layers with 100, 50, and 20 units, respectively, are used. AB–CD–E model uses a dropout rate of 0.1, while AB–C–D–E and A–B–C–D–E models use a dropout rate of 0.25.

A–E and B–E classification models were trained for 50 epochs, AB–CD–E model for 150 epochs, and the AB–C–D–E and A–B–C–D–E models for 100 epochs.

## 4 Results

In this paper, the performance of the algorithm was estimated using the following statistical metrics:

1. Accuracy.
2. Sensitivity.
3. Specificity.
4. F1 score.

The true positives (TP), true negatives (TN), false positives (FP), and false negatives (FN) are extracted from the confusion matrix. The Equations 1–4 indicate the calculation of the performance metrics used in this study.


(1) $$\begin{array}{l} Accuracy=\frac{TP+TN}{TP+TN+FP+FN} \end{array}$$

(2) $$\begin{array}{l} Sensitivity=\frac{TP}{FN+TP} \end{array}$$

(3) $$\begin{array}{l} Specificity=\frac{TN}{FP+TN} \end{array}$$

(4) $$\begin{array}{l} F1\;Score=\frac{2TP}{2TP+FP+FN} \end{array}$$

The accuracy, specificity, sensitivity and F1 score was calculated for each iteration of training for each classification in the study and the average values are reported in the paper.

Table 3 summarizes the results obtained from the experiments.

**Table 3:** Performance metrics obtained.

**Study**	**Classification**	**Model**	**Accuracy (%)**	**Specificity (%)**	**Sensitivity (%)**	**F1 score**
Study 1	A–E	LSTM	100	100	100	100
		GRU	100	100	100	100
	B–E	LSTM	100	100	100	100
		GRU	100	100	100	100
	AB–CD–E	LSTM	98.4	99.12	98.36	98.49
		GRU	98.8	99.32	98.78	98.89
	AB–C–D–E	LSTM	97.8	99.19	97.37	97.63
		GRU	98.4	99.47	98.09	98.09
	A–B–C–D–E	LSTM	97.75	99.39	97.6	97.50
		GRU	98.18	99.49	98	97.94
Study 2	A–E	LSTM	100	100	100	100
		GRU	100	100	100	100
	B–E	LSTM	100	100	100	100
		GRU	100	100	100	100
	AB–CD–E	LSTM	98.4	99.14	98.35	98.40
		GRU	98	98.94	97.91	97.86
	AB–C–D–E	LSTM	97	99.016	96.69	96.42
		GRU	98	99.31	97.44	97.57
	A–B–C–D–E	LSTM	97.8	99.43	97.77	97.65
		GRU	97.8	97.8	97.77	97.79
Study 3	A–E	LSTM	100	100	100	100
		GRU	100	100	100	100
	B–E	LSTM	100	100	100	100
		GRU	100	100	100	100
	AB–CD–E	LSTM	98.1	98.99	98.08	98.13
		GRU	99.2	99.55	99.14	99.23
	AB–C–D–E	LSTM	97	98.94	96.69	96.56
		GRU	97	98.95	96.51	96.48
	A–B–C–D–E	LSTM	96.67	99.14	96.67	96.54
		GRU	96.2	99.03	96.08	96.01
Study 4	A–E	LSTM	99.75	99.75	99.75	99.75
		GRU	99.75	99.75	99.75	99.75
	B–E	LSTM	100	100	100	100
		GRU	99.75	99.7	99.74	99.75
	AB–CD–E	LSTM	97.9	98.85	98.22	98.22
		GRU	97.2	98.44	97.64	97.54
	AB–C–D–E	LSTM	97.7	99.20	97.18	97.32
		GRU	96.8	98.95	96.12	96.004
	A–B–C–D–E	LSTM	97.3	99.29	97.29	97.18
		GRU	97	99.22	97	96.94
Study 5	A–E	LSTM	99.17	99.17	99.17	99.16
		GRU	99.05	99.05	99.05	99.04
	B–E	LSTM	99	99	99	98.999
		GRU	99.03	99.03	99.03	99.02
	AB–CD–E	LSTM	98.09	98.94	98.124	98.21
		GRU	98.44	99.13	98.42	98.53
	AB–C–D–E	LSTM	96.78	98.88	96.027	96.07
		GRU	96.62	98.80	95.78	95.86
	A–B–C–D–E	LSTM	95.50	98.75	95.50	95.5
		GRU	95.8	98.81	95.80	95.79
Study 6	A–E	LSTM	99.89	99.89	99.88	99.89
		GRU	99.91	99.91	99.91	99.91
	B–E	LSTM	99.43	99.43	99.43	99.43
		GRU	99.5	99.49	99.49	99.50
	AB–CD–E	LSTM	98.28	99.05	98.41	98.458
		GRU	97.9	98.84	97.9	98.02
	AB–C–D–E	LSTM	96.82	98.88	96.27	96.33
		GRU	96.58	98.80	96.03	96.06
	A–B–C–D–E	LSTM	95.56	98.76	95.56	95.57
		GRU	95.8	98.81	95.8	95.8

The average of the training times for each iteration has been reported in this paper. The inference times of the test sets for all the classifications in each study are listed in Table 4.

**Table 4:** Inference time(s).

**Study type**		**A–E**	**B–E**	**AB–CD–E**	**AB–C–D–E**	**A–B–C–D–E**
Study 1.1	Mean inference time(s)	0.49	0.32	0.96	0.91	0.58
	Mean training time(s)	77.63	138.82	394.01	455.29	412.68
Study 1.2	Mean inference time(s)	0.37	0.33	0.81	0.72	0.64
	Mean training time(s)	74.02	127.68	739.45	505.55	505.12
Study 2.1	Mean inference time(s)	0.31	0.32	0.61	0.76	0.94
	Mean training time(s)	254.09	266.86	383.81	495.88	622.77
Study 2.2	Mean inference time(s)	0.35	0.56	0.94	0.67	0.69
	Mean training time(s)	63.75	130.85	620.25	439.84	435.87
Study 3.1	Mean inference time(s)	0.53	0.53	0.92	0.86	0.92
	Mean training time(s)	129.36	132.92	192.10	269.83	456.04
Study 3.2	Mean inference time(s)	0.28	0.32	1.28	0.60	0.66
	Mean training set(s)	66.6	64.16	618.95	154.11	335.2

## 5 Discussion

In this section, we compare the performance metrics achieved by our framework with the works by other authors for epileptic seizure detection, based on the common performance metrics.

Studies 1, 2, and 3, across binary classification A–E, achieve perfect 100% accuracy. Study 4 reports an accuracy of 99.75%, while Study 5.1 and 6.2 achieve remarkable accuracies of 99.16 and 99.91% respectively, for A–E classification. These metrics are comparable with the latest state-of-art methods as shown in the Table 5.

**Table 5:** Performance metrics achieved by other works for classification between A–E.

**Author**	**Method**	**Accuracy**	**Specificity**	**Sensitivity**
Tzallas et al. (2007)	Time-frequency analysis and ANN	100%	100%	100%
Ghosh-Dastidar and Adeli (2009)	Levenberg-Marquardt backpropagation neural network	96.7%	–	–
Chandaka et al. (2009)	Cross-correlation aided SVM	95.96%	–	–
Chua et al. (2011)	Gaussian mixture model	93.1%	94.8%	89.7%
Orhan et al. (2011)	K-means clustering and MLP neural network model	100%	100%	100$
Kaya et al. (2014)	1D LBP and functional tree	97.50%	99%	96%
Samiee et al. (2014)	Rational discrete STFT and MLP classifier	99.80%	–	–
Peker et al. (2015)	Dual tree complex wavelet transform + complex-valued neural networks	100%	100%	100%
Bhattacharyya et al. (2017)	TQWT-based multi-scale K-NN entropy	100%	100%	100%
Vipani et al. (2017)	Hilbert transform	89.31%	–	–
Singh and Dehuri (2019)	DWT+ MLP neural network	99.50%	–	–
Mamli and Kalbkhani (2019)	Fourier synchro-squeezed transform+gray-level co-occurrence matrix+SVM	100%	–	–
Thara et al. (2019)	Deep neural networks	97.21%	91.47%	98.59%
Raghu et al. (2019)	Matrix determinant + MLP	99.45%	–	–
Akyol (2020)	Stacking ensemble based deep learning approach	97.17%	98.18%	93.11%
Varlı and Yılmaz (2023)	Combined learning using CWT	99.07%	–	–
Varlı and Yılmaz (2023)	Combined learning using STFT	99.28%	–	–
Proposed framework	Study 1.1, 1.2, 2.1, 2.2, 3.1, 3.2	**100%**	**100%**	**100%**
	Study 4.1, 4.2	99.75%	99.75%	99.75%
	Study 5.1	99.17%	99.17%	99.17%
	Study 5.2	99.05%	99.05%	99.05%
	Study 6.1	99.89%	99.89%	99.88%
	Study 6.2	99.91%	99.91%	99.91%

The values in bold are the highest values we have obtained in the study.

For binary classification B–E, Studies 1, 2, 3, and 4.1 show perfect accuracy, Study 5.1 achieves 99%, Study 5.2 achieves 99.02%, and Study 6 achieves the highest accuracy of 99.5%. The comparison of our work with the previous works has been given in Table 6.

**Table 6:** Performance metrics achieved by other works for classification between normal and seizure waveforms: B vs. E.

**Author**	**Method**	**Accuracy**	**Specificity**	**Sensitivity**
Samiee et al. (2014)	Rational discrete STFT and MLP classifier	99.30%	–	–
Bhattacharyya et al. (2017)	TQWT-based multi-scale K-NN entropy	99.5%	99%	100%
Singh and Dehuri (2019)	DWT + multilayer perceptron neural network	97.00%	–	–
Mamli and Kalbkhani (2019)	Fourier synchro-squeezed transform+gray-level co-occurrence matrix+SVM	99.38%	–	–
Thara et al. (2019)	Deep neural networks	97.21%	91.47%	98.59%
Raghu et al. (2019)	Matrix determinant + MLP	96.06%	–	–
Akyol (2020)	Stacking ensemble based deep learning approach	97.17%	98.18%	93.11%
Proposed framework	Study 1.1, Study 1.2, Study 2.1, Study 2.2, Study 3.1, Study 3.2, Study 4.1	**100%**	**100%**	**100%**
	Study 4.2	99.75%	99.75%	99.75%
	Study 5.1	99%	99%	99%
	Study 5.2	99.03%	99.03%	99.03%
	Study 6.1	99.43%	99.43%	99.43%
	Study 6.2	99.5%	99.49%	99.49%

The values in bold are the highest values we have obtained in the study.

Table 7 compares the best performance metric obtained by our work with the other latest works in the field of ternary classification.

**Table 7:** Performance metrics achieved by other works for classification between normal-ictal-interictal waveforms.

**Author**	**Method**	**Accuracy**	**Specificity**	**Sensitivity**
Tzallas et al. (2007)	Time-frequency analysis	97.72%	98.75%	96.93%
Orhan et al. (2011)	K-means clustering and multilayer perceptron neural network model	95.60%	97.64%	90.51%
Acharya et al. (2012)	Fuzzy Sugeno	96.7%	99%	95%
Peker et al. (2015)	Dual tree complex wavelet transform and complex-valued neural networks	98.28%	–	–
Bhattacharyya et al. (2017)	TQWT-based multi-scale K-NN entropy	98.60%	98.67%	98.5%
Tiwari et al. (2017)	Keypoint-based LBP + SVM	98.80%	–	–
Acharya et al. (2018)	Deep convolutional neural networks	88.7%	90%	95%
Raghu et al. (2019)	Matrix determinant + MLP	96.50%	–	–
Gupta and Pachori (2019)	FBSE + WMRPE + Regression	98.6%	–	–
Miltiadous et al. (2021)	Discrete wavelet transform + Random forest classifier	95.84%	97.75%	96.04%
Abiyev et al. (2020)	CNN (10 fold cross-validation)	98.6%	98.83%	97.67%
Zhao et al. (2020)	1D-CNN + batch normalization	96.97%	–	–
Hassan et al. (2022)	1D-CNN + Bagged KNN	99%	–	–
Proposed framework	Study 1.1	98.4%	99.12%	98.36%
	Study 1.2	98.8%	99.32%	98.78%
	Study 2.1	98.4%	99.14%	98.35%
	Study 2.2	98%	98.94%	97.91%
	Study 3.1	98.1%	98.99%	98.08%
	Study 3.2	**99.2%**	**99.55%**	**99.14%**
	Study 4.1	97.9%	98.85%	98.22%
	Study 4.2	97.2%	98.44%	97.64%
	Study 5.1	98.09%	98.94%	98.124%
	Study 5.2	98.44%	99.13%	98.42%
	Study 6.1	98.28%	99.05%	98.41%
	Study 6.2	97.9%	98.84%	97.9%

The values in bold are the highest values we have obtained in the study.

For the three-class classification between AB–CD–E(healthy-interictal-ictal), Study 3.2 achieves the highest accuracy of 99.2%, specificity of 99.55%, sensitivity of 99.14% and F1 score of 99.23%.

For four-class classification, our proposed models outperform the latest works by a margin of 0.58%–to 2.4%. The Tables 8, 9 compare the performance of the best performance metrics obtained by the proposed work with the latest works in the field of the four-class and five-class classification.

**Table 8:** Performance metrics achieved by other works for classification between four-class classifications.

**Author**	**Method**	**Classification type**	**Accuracy**	**Specificity**	**Sensitivity**
Türk and Özerdem (2019)	CWT + CNN	B-C-D-E	91.50%	–	–
Hussain and Qaisar (2022)	DWT + MI-based feature selection		96%	–	–
Hassan et al. (2022)	Hybrid 1D-CNN	AB–C–D–E	96%	–	–
Proposed Framework	Study 1.1	AB–C–D–E	97.8%	99.19%	97.37%
	Study 1.2	AB–C–D–E	**98.4%**	**99.47%**	**98.09%**
	Study 2.1	AB–C–D–E	97%	99.016%	96.69%
	Study 2.2	AB–C–D–E	98%	99.31%	97.44%
	Study 3.1	AB–C–D–E	97%	98.94%	96.69%
	Study 3.2	AB–C–D–E	97%	98.95%	96.51%
	Study 4.1	AB–C–D–E	97.7%	99.2%	97.18%
	Study 4.2	AB–C–D–E	96.8%	98.95%	96.12%
	Study 5.1	AB–C–D–E	96.78%	98.88%	96.027%
	Study 5.2	AB–C–D–E	96.62%	98.80%	95.78%
	Study 6.1	AB–C–D–E	96.82%	98.88%	96.27%
	Study 6.2	AB–C–D–E	96.58%	98.80%	96.03%

The values in bold are the highest values we have obtained in the study.

**Table 9:** Performance metrics achieved by other works for classification between five-class classifications.

**Author**	**Method**	**Accuracy**	**Specificity**	**Sensitivity**
Zahra et al. (2017)	Multivariate empirical mode decomposition	87.2%	–	–
Miltiadous et al. (2021)	Discrete wavelet transform + Random forest classifier	82.25%	95%	82.25%
Türk and Özerdem (2019)	CWT + CNN	93.60%	–	–
Zhao et al. (2020)	1D-CNN + Batch-normalization	93.55%	95.93%	83.73%
Hassan et al. (2022)	1D-CNN + SVM	93.6%	–	–
Proposed framework	Study 1.1	97.75%	99.39%	97.6%
	Study 1.2	**98.18%**	**99.49%**	**98%**
	Study 2.1	97.8%	99.43%	97.77%
	Study 2.2	97.8%	97.8%	98.001%
	Study 3.1	96.6%	99.14%	96.67%
	Study 3.2	96.2%	99.03%	96.08%
	Study 4.1	97.3%	99.29%	97.29%
	Study 4.2	97%	99.22%	97%
	Study 5.1	95.5%	98.75%	95.5%
	Study 5.2	95.80%	98.81%	95.80%
	Study 6.1	95.56%	98.76%	95.56%
	Study 6.2	95.8%	98.81%	95.8%

The values in bold are the highest values we have obtained in the study.

Study 1 conducted with unfiltered EEG signals of duration 23.6 s gives the highest classification accuracy into classes A–B–C–D–E with an accuracy of 98%, specificity of 99.49%, 98.18% and F1 score of 97.94%. An improvement by 4% over the model proposed by Türk and Özerdem (2019) and Hassan et al. (2022) is obtained by the Bi-LSTM model of Study 1 and an improvement by 4.4% by the Bi-GRU model of Study 1. Study 2 achieves an accuracy of 97.8% outperforming the best achieved performance by 4.2%. Study 3 achieves an accuracy of 96.6 and 96.2% by the Bi-LSTM and the Bi-GRU models respectively. Study 4 achieves an accuracy of 97.3 and 97% by the Bi-LSTM and Bi-GRU models again outperforming the existing methods.

## 6 Ablation study

In this section, we run a series of ablation experiments to test the effectiveness of each part of the proposed framework. First, we remove all the 1D-CNN layers from the models and re-train each model. In the Study 1, for the A–E classification, the accuracy decreased from 100 to 99%. The specificity and sensitivity also showed a drop to 98.82%. Similarly, for the B–E classification, the accuracy dropped to 99%. For the AB–CD–E classification, the accuracy showed a sharp drop to 93.59%, whereas the accuracy of the proposed model shows an accuracy of 98.4%. A drop of ~2.67% in specificity and 4.58% in sensitivity is also noted. On removal of 1D-CNN layers, a 1.8% drop in the accuracy is seen in the AB–C–D–E classification and a drop of 1.2% in the A–B–C–D–E classification. The ablation study in Study 2 shows a drop of 1.5% and 1% in the accuracies for the A–E and B–E classifications, respectively. The AB–CD–E classification results show a decrease in accuracy by 3.2%. The ablation study for AB–C–D–E showed a decrease in accuracy by 1% and the A–B–C–D–E classification showed a decrease by 0.2%.

The ablation study in Study 3 results in a decrease in the accuracy of the AB–CD–E classification by 1.9%, in the accuracy of AB–C–D–E classification by 0.6% and in A–B–C–D–E classification by 2.2%. The A–E and B–E classifications also result in a drop in accuracy by 1.5 and 1%, respectively. The ablation study in Study 4 for the A–B–C–D–E classification shows a drop in accuracy by 1.9%. The sensitivity in the AB–CD–E classification decreases by 0.32%.

In Study 5, there is a drop in accuracy to 97.56% from 99.17% for the A–E classification and a drop in accuracy to 98.54% from 99% for the B–E classification. On removal of the 1D-CNN layers, we see a drop of accuracy in the AB–CD–E classification to 93.94% from 98.09%. Similarly, there is a drop in accuracies for AB–C–D–E and A–B–C–D–E classifications.

In Study 6, for the AB–CD–E classification, the accuracy drops by 5.73% on the removal of all the 1D-CNN layers. Similarly, a drop of 4.07% of accuracy is seen in the AB–C–D–E classification. For the A–B–C–D–E classification, a drop in the performance metrics is observed.

In the next set of experiments for the ablation study, the Bidirectional LSTM layers from the proposed models were removed and then trained again. We have reported the results for the Bi-LSTM models only.

The A–E classification of Study 1 showed a drop in accuracy by 4.5% on the removal of the Bi-LSTM layers. This classification also showed a drop in specificity by 3.92%. The AB–CD–E classification results show a drop of 9% in accuracy, a drop of 5.44% in specificity and a drop of 10.54% in sensitivity on the removal of all the Bi-LSTM layers in the proposed model. Similarly, the AB–C–D–E classification results show a decrease in accuracy to 95%. The A–B–C–D–E classification results show a decrease in accuracy by 8.21% on the removal of Bi-LSTM layers on the removal of Bi-LSTM layers.

In Study 2, the A–E classification results show a decrease in the accuracy to 98.5%. The accuracy for the B–E classification decreases to 94%. A drop of 6% in accuracy is observed. The AB–CD–E classification results show a decrease in the accuracy by 3.3%–95.1%. An accuracy of 94.4% is observed in the case of AB–C–D–E classification which is a decrease of 2.6% from the accuracy observed in the proposed model. The A–B–C–D–E classification results shows a decrease of 2.6% in accuracy.

In Study 3, a drop of accuracy by 10.51% is observed for the A–E classification and a drop of accuracy by 12% for the B–E classification. Similarly, a decrease in performance metrics is observed for the AB–CD–E and AB–C–D–E classifications. The A–B–C–D–E classification results register a decrease of 5.4% in accuracy on the removal of the Bi-LSTM layers.

In Study 4, the accuracies for the A–E and B–E classifications drop to 94.75%. The AB–CD–E classification results register a decrease in accuracy by 8.7%. Similarly, the accuracy drops by 1.4% in the AB–C–D–E classification and by 6.1% for the A–B–C–D–E classification. Similarly, the other performance metrics used for evaluating the performance of the proposed models also show a decrease.

The accuracy for the A–E classification in the Study 5 decreases to 97.52% from 99%. The AB–CD–E classification results show a drop in accuracy by 10.88%–87.21%, while the AB–C–D–E classification results show a decrease in accuracy by 4.75% to 92.027% on the removal of the Bi-LSTM layers. The A–B–C–D–E classification results show a decrease in the accuracy by 2.48%.

In the Study 6, the A–E classification results show a decrease in accuracy by 4.17%, whereas the B–E classification results show a decrease in accuracy by 3.84% on the removal of the Bi-LSTM layers. The accuracy of AB–CD–E classification decreases by 4.28% on the removal of the Bi-LSTM layers. The accuracy for the AB–C–D–E classification decreases to 94.4% and for the A–B–C–D–E classification to 94.12%.

The results obtained from the ablation studies are summarized under Table 10.

**Table 10:** Ablation study.

**Study**	**Classification type**	**Accuracy**	**Specificity**	**Sensitivity**	**F1 score**
**1D-CNN layers**					
Study 1	A–E	99%	98.82%	98.82%	98.95
	B–E	99%	99.11%	99.11%	98.98
	AB–CD–E	93.59%	96.45%	93.78%	93.96
	AB–C–D–E	96%	98.56%	95.64%	95.51
	A–B–C–D–E	96.4%	99.01%	96.335%	96.335
Study 2	A–E	98.5%	98.69%	98.69%	98.49
	B–E	99%	99%	99%	98.98
	AB–CD–E	95.2%	97.4%	94.95%	94.93
	AB–C–D–E	96%	98.5%	95.99%	95.99
	A–B–C–D–E	97.6%	99.3%	97.5%	97.59
Study 3	A–E	98.5%	98.5%	98.5%	98.47
	B–E	99%	99%	99%	98.98
	AB–CD–E	96.5%	98.1%	95.99%	95.87
	AB–C–D–E	96.4%	98.71%	9582%	95.87
	A–B–C–D–E	94.4%	98.5%	94.3%	94.25
Study 4	A–E	99.4%	99.39%	99.39%	99.47
	B–E	99%%	99%	99%	98.98
	AB–CD–E	98.1%	98.9%	97.9%	98.02
	AB–C–D–E	97.7%	99.19%	97.16%	97.25
	A–B–C–D–E	95.4%	98.74%	95.62%	95.41
Study 5	A–E	97.56%	97.55%	97.56%	97.56
	B–E	98.54%	98.53%	98.53%	98.54
	AB–CD–E	93.94%	96.71%	92.75%	92.82
	AB–C–D–E	95.07%	98.21%	94.26%	94.34
	A–B–C–D–E	94.47%	98.39%	94.56%	94.56
Study 6	A–E	99.8%	99.83%	99.83%	99.83
	B–E	99.34%	99.33%	99.33%	99.33
	AB–CD–E	92.55%	95.81%	93.59%	93.59
	AB–C–D–E	92.75%	97.24%	93.06%	92.75
	A–B–C–D–E	95.1%	98.58%	95.15%	95.15
**Bi-LSTM layers**					
Study 1	A–E	95.5%	96.08%	96.08%	95.47
	B–E	94%	94.78%	94.78%	93.86
	AB–CD–E	89.40%	93.68%	87.82%	88.16
	AB–C–D–E	95%	98.05%	94.83%	94.84
	A–B–C–D–E	89.39%	96.82%	89.65%	89.24
Study 2	A–E	98.5%	98.67%	98.67%	98.47
	AB–CD–E	95.1%	97.11%	93.35%	93.79
	AB–C–D–E	94.4%	97.65%	94.33%	94.307
	A–B–C–D–E	95.2%	98.74%	94.97%	94.705
Study 3	A–E	89.49%	89.62%	89.62%	89.42
	B–E	88%	88.2%	88.2%	87.95
	AB–CD–E	98.4%	99.1%	98.5%	98.47
	AB–C–D–E	96.2%	98.62%	95.63%	95.7
	A–B–C–D–E	91.2%	97.2%	91.48%	91.48
Study 4	A–E	94.75%	95.32%	95.32%	94.73
	B–E	94.75%	95.04%	95.04%	94.73
	AB–CD–E	89.2%	93.90%	89.53%	88.83
	AB–C–D–E	96.4%	98.73%	96.11%	96.05
	A–B–C–D–E	90.8%	97.12%	91%	90.77
Study 5	A–E	97.52%	97.49%	97.49%	97.51
	B–E	99.04%	99.04%	99.05%	99.04
	AB–CD–E	87.209%	93.04%	85.81%	85.75
	AB–C–D–E	92.027%	97.01%	90.74%	90.79
	A–B–C–D–E	93.01%	97.9%	93.077%	93.16
Study 6	A–E	95.7%	95.71%	95.71%	95.69
	B–E	95.59%	95.57%	95.57%	95.57
	AB–CD–E	94%	96.6%	94.74%	94.80
	AB–C–D–E	94.4%	97.81%	94.29%	94.41
	A–B–C–D–E	94.12%	98.31%	94.15%	94.15

## 7 Conclusions

This paper introduces a robust and novel framework for detecting epileptic seizures using a combination of 1D-CNN, Bidirectional LSTMs and GRUs, and Average Pooling Layer as a unit. The framework demonstrates its effectiveness in accurately distinguishing between different classes within the Bonn dataset. Extensive evaluations were conducted under both ideal and imperfect conditions, and it was found that the proposed models involving Bidirectional LSTMs and Bidirectional GRUs yield comparable results. The proposed work has obtained significant advancements in the accuracy, specificity and sensitivity obtained from the existing methods for the detection of epileptic seizures, especially 3, 4, and 5 class classifications. Our proposed model achieves an accuracy of 99%–100% for binary classifications into seizure and normal waveforms, 97.2%–99.2% accuracy for ternary classifications, 96.2%–98.4% accuracy for four class classifications, and accuracy of 95.81%–98% for five class classification. Furthermore, the proposed framework proves its efficacy in detecting epileptic seizures within EEG signals of varying durations by varying the parameters used in the proposed models, encompassing both longer and shorter durations. These findings highlight the suitability of the proposed framework for reliable epileptic seizure detection using EEG signals. This work can also be extended to test its efficacy in the classifications for other datasets.

## Data availability statement

The original contributions presented in the study are included in the article/supplementary material, further inquiries can be directed to the corresponding author.

## Author contributions

SM: Conceptualization, Formal Analysis, Methodology, Investigation, Visualization, Software, Writing – original draft, Writing – review & editing. VB: Supervision, Writing – original draft, Writing – review & editing.
